# Response of Yield and Yield Components of *Tef* [*Eragrostis tef* (Zucc.) Trotter] to Tillage, Nutrient, and Weed Management Practices in Dura Area, Northern Ethiopia

**DOI:** 10.1155/2014/439718

**Published:** 2014-07-13

**Authors:** Gebreyesus Brhane Tesfahunegn

**Affiliations:** College of Agriculture, Aksum University-Shire Campus, P.O. Box 314, Shire, Ethiopia

## Abstract

The low average grain yield (0.7 ton ha^−1^) of *tef* in Ethiopia is mainly attributed to low soil fertility, and inappropriate tillage and weeds control practices. Despite this, limited scientific information has been documented so far on their interaction effects on *tef* crop productivity in northern Ethiopia. The objective of this study was to assess the separate and interaction effects of tillage, fertilizer, and weed control practices on *tef* yield and yield components in the conditions of northern Ethiopia. A two-year study (2008-2009) was conducted using split-split-plot design with three replications. In the main plot, three tillage treatments: conventional tillage (6 times tillage passes) (T1), four times tillage passes (T2), and reduced tillage (single tillage pass at sowing) (T3) were applied. The fertilizer treatments in the subplots were: no fertilizer (F1); 23 kg N ha^−1^ (F2); 23 kg N ha^−1^ and 10 kg P ha^−1^ (F3); 23 kg N ha^−1^ and 2.5 ton manure ha^−1^ (F4); and 2.5 ton manure ha^−1^ (F5). The sub-subplot weed control treatments included farmer weed control practice or hand weeding (W1); 2,4 D at 0.75 kg ha^−1^ at five-leaf stage; 2,4 D at 0.75 kg ha^−1^ at six-leaf stage; 2,4 D at 1.5 kg ha^−1^ at five-leaf stage; and 2,4 D at 1.5 kg ha^−1^ at six-leaf stage. This study showed that the separate and interaction effects of tillage, fertilizer, and weed control practices significantly affected *tef* crop yield and yield components in both crop seasons. T2 increased *tef* yield by >42% over the other tillage and F3 increased yield by >21% over the other fertilizer treatments. Grain yield increased by >23% due to W1. This study thus suggested that promising treatments such as T2, F3, and W1 should be demonstrated at on-farm fields in order to evaluate their performance at farmers' conditions.

## 1. Introduction

*Tef* [*Eragrostis tef* (Zucc.) Trotter] is an Ethiopian cultivated crop which is mainly grown for its grain as a major staple food and market cereal crop. Most* tef* grains are made into* injera* (most popular food in the national diet), which is flat spongy and slightly sour bread that looks like a giant bubbly pancake [1]. The annual area covered by* tef* during the main season is about 1.91 million ha, that is, accounted for 29% of the total cereal area in Ethiopia which stands first in its area coverage [2]. Despite the fact that* tef* has widely grown under a wide range of altitudes (300 to 2800 m above sea level), climate conditions, and soil types [1, 3], the average grain yield in Ethiopia is about 0.7 ton ha^−1^ [3]. A lower* tef* yield than this could be found in the dry parts of the country [3].

Lower* tef* grain yield is mainly attributed to low soil fertility, especially, nitrogen (N) and phosphorus (P) deficiencies [4] and inappropriate tillage and weed control practices [3, 5]. Other studies also added that weed is one of the key limiting factors for attaining higher* tef* yield [6]. Despite this fact, limited research information has documented so far the interaction effects of tillage practices and organic and inorganic fertilizers integrated with weed management practices on yield and yield component of* tef* crop in northern Ethiopia.

Low soil fertility is exacerbated by soil fertility depletion through nutrient removal with harvest, tillage, weeding, and losses in runoff and soil erosion [7, 8]. Many farmers are unable to compensate for such losses, which resulted in negative nutrient balances [9]. This situation is worsened by low input continuous cultivation and overgrazing [7]. Researchers suggested that nutrient availability can be improved by nutrient application such as inorganic or organic fertilizer or their combination (e.g., [10]). Despite the great efforts on N and P sources fertilizers application to improve the yield of* tef* by farmers, the nonaffordable cost of inorganic fertilizer and high risk in the semiarid areas challenge adopting fertilizer technology by farmers.

Substantial studies have shown that intensive cultivation generally increases the potential for soil erosion due to the breakdown of soil aggregates and reduction of soil cohesion and thus decreases soil nutrient (e.g., [11, 12]). Generally, farmers practice with conventional tillage is aimed to control weeds, conserve moisture, and increase soil warming [5]. But the consequence of such repeated operations causes moist soil to move to the surface which favors loss of water by evaporation, exposing soil to erosion damage [5]. Many other studies have also noted that when a similar tillage system has been practiced for a long period, the practice can alter soil properties and agricultural production [13–17]. Conventional tillage is reported as a factor that aggravates crop yield reduction [3, 18, 19], but it is still continuing as sole cultivation method in northern Ethiopia. In contrast to this, other studies have reported a significant increase in crop productivity using reduced tillage [20, 21]. Such increment could be a result of its long-term effect on an overall hydrological behaviour of soils [13, 17, 20]. Other researchers (e.g., [22–24]) noted that it is essential to select a tillage practice that sustains and favors successful growth of agricultural crops in a given environmental condition.

The capacity of* tef* to compete with weeds, which is one of the main yield limiting biotic factors, is poor. As a result, hand weeding as the main method of weed control has been practiced for many years even though labor shortage as a constraint is being increased. In view of such shortcoming, herbicides (e.g., 2,4D and MCPA) have been applied on* tef* even though the rate and time of application varied across the smallholder farmers [25]. It is clearly indicated in the previous studies that emphasis should be given to both rate and time of application of herbicides on* tef* crop [25, 26]. This is because the response of cereals such as* tef* to the herbicides varies with growth (leaf) stages as herbicides can cause leaf deformity and sterility at certain stages [25–27], indicating that identification of the safest time of application (most tolerance growth stage) is crucial.

Even though variability in tillage types and intensity, fertilizer rates and types, and weed control practices can affect crop production, limited scientific information is documented with regard to their short-term effects on* tef* crop production in a Cambisols in northern Ethiopia. However, it is a prerequisite to quantify such effects on crop production while designing site-specific applications such as fertilizer rates, proper tillage frequency, and weed management practices [11]. This study was aimed to assess* tef *yield and yield components response to separation and interaction effects of tillage, fertilizer, and weed control practices.

## 2. Materials and Methods

### 2.1. Study Area Description

The experiment was conducted under rain-fed conditions from 2008 to 2009 (two years) at Dura Farmers Training Center, an administrative unit of Tigray region of northern Ethiopia. The location of the study site is latitude: 14°07′N, longitude: 38°44′E, and altitude: 2050 m a. s. l. The slope of the experimental site was about 2%. The site is one of the most* tef* growing areas in the Tigray region but productivity has hampered by different factors. It has a unimodal rainfall with the main rainy season from July to early September. More than 68% of the annual rainfall was recorded in July and August. The average annual rainfall and temperature of the area are 700 mm and 20°C, respectively.

**Figure 1:**
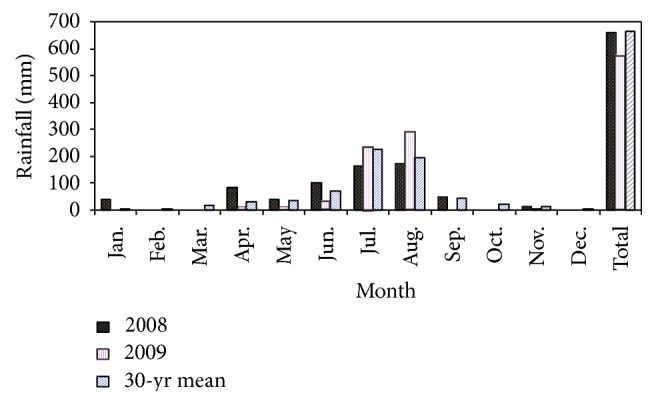
Monthly and total annual rainfall for 2008, 2009, and 30-year average (source: Meteorological agency, Mekelle branch).

The rainfall during planting time (July 2008) was lower as compared to the 30-year average. However, the rainfall during July 2009 (planting time) and the 30-year average was almost the same in amount (Figure 1). A higher rainfall in August 2009 as compared to August 2008 and the 30-year average was observed. Despite this, during the grain filling stage of most crops (September 2009), rainfall was little to none. The reverse was observed in September 2008 and for the 30-year average (Figure 1). This figure also showed a similar trend in total annual rainfall in 2008 and the 30-year average.

The soil texture of the experimental site (49% sand, 25% silt, and 26% clay) was sandy clay loam and the soil type is classified as Chromic Cambisols according to FAO/UNESCO [28]. The soil showed neutral pH (7.07), very low organic carbon (0.76%), low total nitrogen (0.05), and medium available phosphorus (8.6 mg kg^−1^). The soil sampling and analysis procedure for these parameters is given briefly in Section 2.3.

### 2.2. Experimental Design, Treatments, and Procedure

The experiment was conducted in 2008 and 2009 crop seasons in which treatments were applied to the same experimental units each year using split-split-plot design with three replications. In the main plot, three tillage treatments in a randomized complete block design (RCBD) were applied. These tillage treatments were conventional tillage (6 times tillage passes) (T1), four times tillage passes (T2), and reduced tillage (single tillage pass at sowing) (T3). The treatments were practiced according to the traditional plowing system of oxen-drawn plow using local implement which is known as* maresha*. The only difference in the treatments was the number of tillage passes.

The fertilizer treatments in the subplots were no fertilizer (F1); 23 kg N ha^−1^ (F2); 23 kg N ha^−1^ and 10 kg P ha^−1^ (F3); 23 kg N ha^−1^ and 2.5 ton manure ha^−1^ (F4); and 2.5 ton manure ha^−1^ (F5). The source of nitrogen (N) was urea and that of phosphorous (P) was diammonium phosphate (DAP). DAP was applied at planting time whereas urea was applied at 4 weeks after planting. Manure was applied just at planting time and mixed with the soil using oxen-plowing. Manure was collected and stored in a pit for 3 months before the application time. The source of manure was cattle which fed indoor commonly on hay and crop residue (mainly* tef* straw) with silage. The literature shows that 2.5 ton manure can produce about 46 kg N but half (50%) of this N was assumed to be exposed to different losses during collection, storage, and application (e.g., Hati et al. [29]).

The sub-subplot weed control treatments were farmer weed control practice (hand weeding) (W1); 2,4D at 0.75 kg ha^−1^ at five-leaf stage (W2); 2,4D at 0.75 kg ha^−1^ at six-leaf stage (W3); 2,4D at 1.5 kg ha^−1^ at five-leaf stage (W4); and 2,4D at 1.5 kg ha^−1^ at six-leaf stage (W5). 2,4D was applied for in season broad leaf control. The* tef* variety DZ/01/974 seeds were broadcasted uniformly by hand at a rate of 30 kg ha^−1^ after plots were compacted by trampling with human labor. All sub-subplots with W1 were weeded manually twice and the remaining weeded one time for narrow leaf weeds only during the cropping season. Dates of all management practices such as planting, weeding, tillage, harvesting, fertilizer, and herbicide application time were recorded (Table 1). The size of the main plot, subplot, and sub-subplot was 19 m × 19 m, 3 m × 19 m, and 3 m × 3 m, respectively. The spacing between main plots, subplots, and sub-subplots was 1.5, 1.0, and 0.5 m, respectively.

### 2.3. Soil Sampling and Analysis

Five composite disturbed soil samples were collected randomly at the 0–20 cm soil depth from the entire experimental site before imposing any treatment at the beginning of the research period in 2008. The disturbed soil samples were pooled and mixed thoroughly in a basket and a subsample of 500 g was taken for analysis. The subsamples were air-dried, passed through 2 mm sieve, and analyzed for selected soil parameters (soil texture, pH, organic carbon, total nitrogen, and available phosphorous) using standard laboratory procedures. Results of soil parameters are described on Section 2.1.

### 2.4. Data Collection and Analysis

Plant height of the* tef* crop was measured from the surface ground to the tip of the panicle at maturity time from representative 10 plants (short, medium, and tall). Tillering of the crop that considered the number of additional plants growing from the main stem of* tef* at each sub-subplot was counted from 10 plants.* Tef* plants in a 1 m^2^ quadrant were harvested for recording above ground biomass and grain yield at full maturity stage from each sub-subplot. A sample of 50 gm seeds of* tef* was weighed and then air-dried and finally adjusted to constant moisture level of 12%. Harvest index was calculated by the ratio of grain yield to above ground biomass. Data were subjected to analyses of variance using Statistix V9 (Analytical Software, Tallahassee, FL). When treatment effects were significant, least significant difference (LSD) mean separation at probability level (*P*) = 0.05 was applied.

## 3. Results and Discussion

### 3.1. Effects of Tillage, Fertilizer, and Weed Control Practices on* Tef* Yield and Yield Components in 2008 Crop Season

The separate and interaction effects of tillage, fertilizer, and weed control practices significantly (*P* ≤ 0.05) affected most yield and yield components of* tef* crop (Table 2). For instance, tiller number was significantly varied between T2 and T3 and T1 and T2. But tiller number was nonsignificantly differed between T1 and T3 (*P* > 0.05), indicating that both treatments did show similar effect on soil moisture and thereby on plant tillering. The highest mean tiller number was observed from T2 (5.6) and the lowest was from T3 (4.3). Tillering was also significantly influenced by fertilizer rate and weed control practices. The unfertilized (F1) plot showed a significantly lower tillering number as compared to the others. However, there were nonsignificant differences in tiller number among the remaining plots which received different fertilizer rates. In general, the effect of fertilizer showed a higher tiller number as compared to the tillage and weed control treatment effects.

A significant difference in* tef* plant height among the tillage, fertilizer, and weed control treatments was observed (Table 2). The tallest plant height was recorded as a result of F3 (102 cm) followed by T2 (98 cm) and W1 (93 cm). The lowest plant height was measured from F1 (68 cm). Plant height influences above ground biomass yield of a crop. Similarly, panicle length and aboveground biomass yield were significantly affected by the separate effects of the treatments (Table 2). The largest* tef* above ground biomass yield was measured from F3 (7.2 t ha^−1^) followed by F4 (6.3 t ha^−1^) whereas the lowest was observed from F1 (4.0 t ha^−1^). This trend was similar with grain yield in which the highest grain yield was measured from F3 (1.7 t ha^−1^) and the lowest was from F1 (0.82 t ha^−1^). The highest HI was found from W1 (0.30). Even though the manure in F4 contains more nutrients, their availability (releasing) to the crop in a season is too slow. In addition, as soil nutrients increase more soil water is demanded by the crop in which water is a critical constraint in the study area conditions. This could be the reason for F4 not having the highest yield as compared to F3. The interaction effects of T × F, T × W, F × W, and T × F × W significantly influenced tillering, plant height, panicle length, biomass yield, grain yield, and HI of the* tef* crop variety tested in this experiment (Table 2). But the influence of the 3-way (T × F × W) interaction on yield and yield components of* tef* was found much better than the 2-way interaction.

### 3.2. Effects of Tillage, Fertilizer, and Weed Control Practices on* Tef* Yield and Yield Components in 2009 Crop Season

The measured agronomic variables of* tef* crop in 2009 crop season showed significant differences for the separate and interaction effects of tillage, fertilizer, and weed control treatments (Table 3). Tillering was significantly influenced by the separate and interaction effects of these treatments. The effect of fertilizer followed by weed control practices showed a higher tiller number. The fertilizer rates that showed the highest tiller number were F3 (6.9) and F4 (6.8). This was followed by the weed control treatment of W1 (6.7). Among the tillage practices, T2 showed the highest tiller number (6.2). The lowest tiller number was measured from F1. The tallest plant height was measured from F4 (105 cm) followed by F3 (104 cm) and T2 (102 cm). From the weed control treatments, W1 resulted in the highest* tef* plant height. The highest panicle length was recorded from the main plot effect of T2 (24 cm) followed by the fertilizer rate of F3 (23 cm). This indicated that panicle length also affected by synergetic effects of tillage practices and soil nutrient additions from fertilizer.

Tillage, fertilizer, and weed control treatments significantly affected aboveground biomass yield of* tef* crop. A higher aboveground biomass yield of* tef* was found from T2. However, the effect of the fertilizer rates of F3 and F4 showed the highest biomass yield. The lowest biomass yield was measured from F1 treatment. Generally, this study showed that the response of aboveground biomass yield to the effects of weed control practices is lower than the separate effects of the other factors (Table 3).* Tef* grain yield was significantly higher in T2 as compared to the other tillage treatments which could be attributed to its contribution in improving soil moisture and nutrient availability. The effects of F3 and F4 on grain yield were significantly higher than the other fertilizer rates. Generally, the effect of fertilizer on grain yield showed was higher than that of weed control treatments. Despite this, the highest HI was found from the weed control treatment of W1 (0.30) which could be attributed to its effect in producing relatively balanced proportion of biomass and grain yield weight. Overall, HI values were very low. The interaction effects of T × F, T × W, F × W, and T × F × W significantly (*P* ≤ 0.05) influenced yield and yield components of* tef* crop in 2009 crop season even though the 3-way (T × F × W) interaction effects on yield and yield components of* tef* were found much better than the 2-way interaction (Table 3).

### 3.3. Pooled Analysis Results of Treatment Effects (2008-2009)

The two-year pooled data analysis showed that tiller number, plant height, aboveground biomass yield, grain yield, and harvest index were significantly influenced by the separate and interaction treatment effects (Table 4). A higher mean tiller number was found for T2 (5.9) and lower for T1 (4.4). The highest tiller number was measured from F3 (6.8) followed by F4 (6.7). Mean tiller number as a result of W1 was 6.6, which was significantly higher than the other weed control practices. The data of* tef* plant height showed significantly higher due to T2 as compared to the other tillage treatments. The tallest plant height of* tef* crop was measured from F3 (103 cm) and the shortest was from F1 (66 cm). Among the weed control practices, W1 showed a significantly higher plant height.

The pooled analysis result on aboveground biomass yield showed a similar trend to that of plant height and tillering. Treatment T2 (6.0 t ha^−1^) showed a higher biomass yield as compared to that of T1 (4.9 t ha^−1^) and T3 (5.3 t ha^−1^). The treatment F3 (7.4 t ha^−1^) showed the highest mean biomass yield as compared to the tillage and weed control treatments. The lowest mean biomass yield was found from W5 (3.6 t ha^−1^) followed by W4 and F1 (3.9 t ha^−1^) which could be most probably due to chemical effects on the crop growth and lack of soil nutrients such as N and P and soil moisture. Nitrogen (N) might not be mineralized at enough level from soil organic matter to meet much of the N needed for the crop unless applied as fertilizer in the study area condition. This study also demonstrated that pooled* tef* grain yield was significantly higher for T2 than T1 and T3. This may be due to poor infiltration rate of the soils treated by T1 and T3 as the time for infiltration could be too short. In such conditions, crop can be exposed to moisture stress especially at establishment and grain-filling stages in which these affect the yield of the crop.

Among the fertilizer rates, F3 (1.70 t ha^−1^) followed by F4 (1.40 t ha^−1^) showed a significantly higher mean grain yield. The greater response to N and P fertilizer was probably because of soil moisture improvement by tillage treatments such as T2, which was the major yield-limiting factor in dry areas. The N rate in F3 was within the recommended average rates of N (40–80 kg N ha^−1^) for* tef* crop [3, 30]. From the weed control practices a higher mean grain yield was found using W1 (1.60 t ha^−1^) followed by W2 (1.30 t ha^−1^). The effect of the chemical weed control rates (broad leaf weeds) on grain yield of* tef* did show poor performance as compared to hand weeding (W1) (Table 4), but cost-benefit analysis should be done to assess their feasibility. The reason for higher yield due to W1 could be associated with the fact that W1 is characterized by removing weeds from their root (uprooting) and this action can increase soil water infiltration. In addition, chemical weed control can harm the growth of the crop if chemicals are not applied on the appropriate growth stage of the crop. The effect of the treatments on HI showed a similar trend to that of biomass and grain yields, but pooled HI values were generally found to be very low. This could be attributed to the disproportionate in weights between grain yield and aboveground biomass yield which might be constrained by poor soil moisture and nutrients availability starting on the establishment and from the booting to grain filling stages of the crop.

### 3.4. Implication of Treatment Effects as Compared to Previous Studies

The study during 2008 crop season demonstrated that as tillage frequency increases, there is a possibility of increasing* tef* yield. This is consistent with the finding in Fufa et al. [31], who reported that increasing tillage frequency increased* tef* yield and increasing fertilizer level to certain point can increase yield and yield component of* tef* crop [3]. The report by Hati et al. [29] showed that frequent plowing controls weed growth which is the cause for about 35% of* tef* yield losses. The report in Rao et al. [32] also indicated that conventional tillage is superior to reduced tillage with crops such as barley (*Hordeum vulgare*) and chickpea (*Cicer arietinum*) grown under semiarid conditions in India.

In contrast to the above results, Aberra [33] reported that in the presence of proper weed management practices plowing more than once may not be necessary even for* tef* fields. In addition, frequent tillage incurs labor cost which is the critical problem of farmers who have no oxen and pay money for a farmer plowing their land [34]. In such situation, there should be a tillage system that reduces the effects of erosion and weed on soil nutrient and moisture availability of* tef* crop yield. Generally, soil infiltration, soil water holding capacity, and rainfall distribution and amount should be taken in account before deciding tillage frequency, because high tillage frequency can increase soil erosion which finally leads to yield reduction [35].

Studies by Aune et al. [20] and Kassie et al. [21] reported that reduced tillage increased crop productivity as compared to the traditional tillage system (about 6 times) and fertilized as compared to nonfertilized fields. These are consistent with the present finding in 2009 crop season but contradicted with the yield and yield components measured in 2008 crop season, indicating that reduced tillage improves the soil system (e.g., soil carbon sequestration, soil nutrients, and soil water holding capacity) and thereby crop productivity after some years as compared to traditional tillage [20, 36]. Despite these facts, the short-term effect of four times tillage passes (T2) showed better yield and yield components of* tef* crop as compared to the other main treatments. This indicates that this is the optimum frequency of plowing in order to achieve the highest yield in the condition of the study site. In agreement to this result, the research in Mekelle (Ethiopia) by Habtegebrial et al. [3] showed a higher* tef* dry-matter, yield, and harvest index with four plowings using traditional plow when compared to minimum tillage even though there were not statistically different. However, their research did not evaluate the effect of more than four times tillage passes such as T1 which was included in the present study. Generally, four times tillage passes (T2) in the present study showed a higher (>60% increment) grain yield of* tef* as compared to the result reported by Habtegebrial et al. [3]. Such variability could be related to factors such as soil type, climate condition, cropping system, and crop and soil management practices.

Other researchers also reported that on some soils and under certain climatic conditions reduced tillage was found to be superior and more cost effective farming practice than conventional tillage [34, 35]. In the literature, however, the economics of the tillage inputs and outputs is not considered, such as energy and labor costs as well as capital investment in equipment and the resultant effect on soils and production, both in the short- and long-term. Thus, research on tillage should address comprehensively the socioeconomic, physical, chemical, and biological effects.

Regardless of the importance of tillage as weed control practice, the application of herbicides by smallholder farmers is being increased from time to time to replace partly or wholly the dependence on hand weeding (manually) of weed control [26, 27]. Because hand weeding is labor intensive and so is difficult to apply on time. Despite this, hand weeding can contribute to increase soil infiltration and not harm crop growth as in the case of chemicals. However, the intention of using chemicals by farmers is increasing from time to time even though there are some concerns such as dose and time of application on different crop types [25, 36]. In this study, 2,4D at 0.75 kg ha^−1^ at five-leaf stage (W2) performed better than the other herbicide treatments. This is consistent with the finding reported by Mersie and Parker [25] and Friesen and Olson [37]; that is,* tef* is tolerant to such herbicide rate at five-leaf stage. The report by Mersie and Parker [25] on the effect of 2,4D applied at the three- and four-leaf stages of* tef* showed leaf and stem deformities at both rates even though these authors did not determine grain yield reduction that resulted from such effects. The report by Friesen and Olson [37] also showed that 2,4D sprayed at the later stage of the crop may cause sterility. This study thus suggests that W2 seems to be optimum for* tef* crop weed control in the study area conditions.

## 4. Conclusion

This study demonstrated that the separate and interaction effects of tillage, fertilizer, and weed control treatments significantly affected the yield and yield components of* tef* crop. The four times tillage passes (T2) showed better yield and yield components as compared to the others. Similarly, the fertilizer rate of 23 kg N ha^−1^ and 10 kg P ha^−1^ (F3) and 23 kg N ha^−1^ and 2.5 t ha^−1^ manure (F4) showed significantly higher yield of* tef* crop. Hand weeding (W1) resulted in a significantly better yield and yield component of* tef* crop as compared to the others. This suggests that there is a need for selecting herbicides that kill narrow leaf grasses to replace hand weeding and understanding of their effects across different growth stages, soil types, and climatic conditions. On the basis of this study it is suggested to demonstrate the promising treatments such as T2 and T3 with F3 and F4 at on-farm fields in order to evaluate their performance at farmers' conditions. In addition, cost-benefit analysis on the promising treatments should be conducted in order to disseminate to users the most viable techniques. In addition, it is suggested that the effect of reduced tillage (T3) on medium and long-term period should be evaluated on* tef* yield and yield components and soil properties dynamics in the condition of Cambisols.

## Figures and Tables

**Table 1:** Agronomic activities of *tef* crop versus their time of application during the experiment period.

Activities	Year	
	2008	2009
1st tillage	05 April 2008	04 April 2009
2nd tillage	04 May 2008	03 May 2009
3rd tillage	02 June 2008	03 June 2009
4th tillage	21 June 2008	20 June 2009
5th tillage	02 July 2008	01 July 2009
Planting time^a^	16 July 2008	15 July 2009
1st hand weeding	14 August 2008	13 August 2009
2,4D application time	15 August 2008	14 August 2009
Urea application time	16 August 2008	15 August 2009
Tillering time	22 August 2008	22 August 2009
2nd hand weeding	02 September 2008	02 September 2009
Stem elongation time	8 September 2008	7 September 2009
Booting stage	21 September 2008	21 September 2008
Grain filling time	15 Oct 2008	15 Oct 2009
Harvesting time	15 November 2008	16 November 2009

^a^The 6th tillage pass for T1, 4th tillage pass for T2, and the 1st tillage pass for T3 were applied just during the planting time.

**Table 2:** Separate and interaction effects of tillage, fertilizer, and weed control treatments on yield and yield components of *tef* crop in 2008 crop season.

Treatment	TL	PH (cm)	PL (cm)	GY (t ha^−1^)	AGB (t ha^−1^)	HI
Tillage (T)						
Conventional tillage (6 times passes) = T1	4.5^b^	84^b^	15^b^	1.2^b^	5.0^b^	0.24^b^
Four times tillage passes = T2	5.6^a^	98^a^	19^a^	1.6^a^	5.7^a^	0.28^a^
Reduced tillage (single tillage pass) = T3	4.3^b^	72^c^	14^b^	1.0^b^	4.8^b^	0.21^c^
Fertilizer (F)						
No fertilizer = F1	4.5^b^	68^c^	14^c^	0.82^d^	4.0^d^	0.21^a^
23 kg N ha^−1^ = F2	6.3^a^	85^b^	18^b^	1.1^bc^	6.2^b^	0.18^b^
23 kg N ha^−1^ and 10 kg P ha^−1^ = F3	6.7^a^	102^a^	21^a^	1.7^a^	7.2^a^	0.24^a^
23 kg N ha^−1^ and 2.5 t ha^−1^ manure = F4	6.5^a^	88^b^	19^b^	1.3^b^	6.3^b^	0.21^a^
2.5 t ha^−1^ manure = F5	6.2^a^	87^b^	18^b^	1.0^c^	5.9^c^	0.17^b^
Weed control practices (W)						
Hand weeding = W1	6.5^a^	93^a^	20^a^	1.6^a^	5.4^a^	0.30^a^
2,4D at 0.75 kg ha^−1^ at five-leaf stage = W2	6.0^a^	88^b^	18^b^	1.3^b^	4.5^b^	0.29^ab^
2,4D at 0.75 kg ha^−1^ at six-leaf stage = W3	5.3^b^	85^b^	15^c^	1.0^c^	4.0^c^	0.25^c^
2,4D at 150 kg ha^−1^ at five-leaf stage = W4	5.5^b^	86^b^	15^c^	1.1^c^	4.1^c^	0.27^bc^
2,4D at 150 kg ha^−1^ at six-leaf stage = W5	4.7^c^	81^c^	13^d^	0.98^c^	3.7^d^	0.26^c^
Interaction						
T × F	∗	∗	∗∗	∗∗	∗∗	∗
T × W	∗	∗	∗	∗	∗	∗
F × W	∗	∗	ns	∗	∗	ns
T × F × W	∗	∗∗	∗∗	∗∗	∗∗	∗

Means within columns with different letters are significantly different at *P* ≤ 0.05.

^*^Significant at the 0.05 probability level; ^**^significant at the 0.01 probability level; ns: nonsignificant at *P* > 0.05.

× indicates interaction; TL: tiller number per plant; PH: plant height; PL: panicle length; GY, grain yield; AGB: above ground biomass yield; HI: harvest index (—).

**Table 3:** Main and interaction effects of tillage, fertilizer, and weed control practices on yield and yield components of *tef* crop in 2009 crop season.

Treatment	TL	PH (cm)	PL (cm)	GY (t ha^−1^)	AGB (t ha^−1^)	HI
Tillage (T)						
Conventional tillage (6 times passes) = T1	4.2^c^	81^c^	13^c^	1.1^c^	4.8^c^	0.23^b^
Four times tillage passes = T2	6.2^a^	101^a^	24^a^	1.7^a^	6.2^a^	0.27^a^
Reduced tillage (single tillage pass) = T3	5.0^b^	94^b^	17^b^	1.4^b^	5.8^b^	0.24^b^
Fertilizer (F)						
No fertilizer = F1	4.0^d^	63^d^	12^c^	0.79^d^	3.7^d^	0.21^bc^
23 kg N ha^−1^ = F2	6.5^b^	88^c^	19^b^	1.1^c^	6.0^c^	0.18^d^
23 kg N ha^−1^ and 10 kg P ha^−1^ = F3	6.9^a^	104^a^	23^a^	1.7^a^	7.5^a^	0.23^a^
23 kg N ha^−1^ and 2.5 t ha^−1^ manure = F4	6.8^a^	105^a^	22^a^	1.5^b^	6.8^b^	0.22^ab^
2.5 t ha^−1^ manure = F5	6.6^b^	98^b^	20^b^	1.2^c^	6.1^c^	0.20^c^
Weed control practices (W)						
Hand weeding = W1	6.7^a^	95^a^	21^a^	1.7^a^	5.6^a^	0.30^a^
2,4D at 0.75 kg ha^−1^ at five-leaf stage = W2	6.2^b^	86^b^	17^b^	1.2^b^	4.3^b^	0.28^b^
2,4D at 0.75 kg ha^−1^ at six-leaf stage = W3	5.3^c^	78^d^	14^c^	0.98^c^	3.9^c^	0.25^c^
2,4D at 150 kg ha^−1^ at five-leaf stage = W4	5.1^c^	82^c^	13^c^	1.0^c^	3.7^c^	0.27^b^
2,4D at 150 kg ha^−1^ at six-leaf stage = W5	4.5^d^	75^e^	11^d^	0.96^c^	3.5^d^	0.27^b^
Interaction						
T × F	∗	∗	∗∗	∗∗	∗∗	∗
T × W	∗	∗	∗	∗	∗	∗
F × W	∗	∗	ns	∗∗	∗	ns
T × F × W	∗∗	∗∗	∗∗	∗∗	∗∗	∗

Means within columns with different letters are significantly different at *P* = 0.05.

^*^Significant at the 0.05 probability level; ^**^significant at the 0.01 probability level; ns: nonsignificant at *P* > 0.05.

× indicates interaction; TL: tiller number per plant; PH: plant height; PL: panicle length; GY: grain yield; AGB: above ground biomass yield; HI: harvest index.

**Table 4:** Pooled data analysis (2008-2009) results of the separate and interaction effects of the treatments on yield and yield components of *tef* crop.

Treatment	TL	PH (cm)	PL (cm)	GY (t ha^−1^)	AGB (t ha^−1^)	HI (%)
Tillage (T)						
Conventional tillage (6 times) passes =T1	4.4^c^	83^b^	14^b^	1.2^b^	4.9^c^	0.24^b^
Four times tillage passes = T2	5.9^a^	100^a^	22^a^	1.7^a^	6.0^a^	0.28^a^
Reduced tillage (single tillage pass) = T3	4.7^b^	83^b^	16^b^	1.2^b^	5.3^b^	0.23^b^
Fertilizer (F)						
No fertilizer = F1	4.3^c^	66^e^	13^c^	0.81^d^	3.9^d^	0.21^b^
23 kg N ha^−1^ = F2	6.4^b^	87^d^	19^b^	1.1^c^	6.1^c^	0.18^c^
23 kg N ha^−1^ and 10 kg P ha^−1^ = F3	6.8^a^	103^a^	22^a^	1.7^a^	7.4^a^	0.23^a^
23 kg N ha^−1^ and 2.5 t ha^−1^ manure = F4	6.7^a^	97^b^	21^a^	1.4^b^	6.6^b^	0.21^b^
2.5 t ha^−1^ manure = F5	6.4^b^	93^b^	19^b^	1.1^c^	6.0^c^	0.18^c^
Weed control practices (W)						
Hand weeding = W1	6.6^a^	94^a^	21^a^	1.6^a^	5.5^a^	0.31^a^
2,4D at 0.75 kg ha^−1^ at five-leaf stage = W2	6.1^b^	87^b^	18^b^	1.3^b^	4.4^b^	0.30^a^
2,4D at 0.75 kg ha^−1^ at six-leaf stage = W3	5.3^c^	82^c^	15^c^	1.0^c^	4.0^c^	0.25^c^
2,4D at 150 kg ha^−1^ at five-leaf stage = W4	5.3^c^	84^c^	14^cd^	1.1^c^	3.9^cd^	0.28^b^
2,4D at 150 kg ha^−1^ at six-leaf stage = W5	4.6^d^	78^d^	12^d^	1.0^c^	3.6^d^	0.28^b^
Interaction						
T × F	∗	∗	∗∗	∗∗	∗∗	∗
T × W	∗	ns	∗	∗	∗	∗
F × W	ns	∗	∗	∗∗	∗	ns
T × F × W	∗∗	∗∗	∗∗	∗∗	∗∗	∗

Means within columns with different letters are significantly different at *P* = 0.05.

^*^Significant at the 0.05 probability level; ^**^significant at the 0.01 probability level; ns: nonsignificant at *P* > 0.05.

× indicates interaction; TL: tiller number per plant counted just before harvesting; PH: plant height; PL: panicle length; GY: grain yield; AGB: above ground biomass yield; HI: harvest index.
